# Outcomes of Biliointestinal Bypass among Iranian Obese Patients

**DOI:** 10.1055/s-0038-1673662

**Published:** 2018-10-18

**Authors:** Seyed Morteza Mousavi Naeini, Alireza Khalaj, Ali Abbaszadeh-Kasbi, Seyed Rouhollah Miri

**Affiliations:** 1 General Surgeon, Private Practice, Tehran, Iran; 2 Department of Surgery, Shahed University of Medical Sciences, Tehran, Iran; 3 School of Medicine, Tehran University of Medical Sciences, Tehran, Iran; 4 Department of Surgery, Tehran University of Medical Sciences, Tehran, Iran

**Keywords:** obesity, BIBP, efficacy, outcome

## Abstract

**Background** There are several surgical approaches to treat obesity not cured with medical approaches. Each method has its advantages and complications. In here, we have conducted a study to evaluate complications of biliointestinal bypass surgery (BIBP).

**Methods** A prospective study was conducted in a private hospital from 2002 to 2016. Those patients, not previously operated for morbid obesity, were eligible. Mean follow-up period was 89 months ( ± 54 months; range: 73–108 months). Main outcome measures were weight, BMI (body mass index), concentrations of blood lipids and glucose, liver transaminases, and obesity-related comorbidities and complications.

**Results** Twenty-three consecutive patients with morbid obesity, including 16 women (69.7%) and seven men (31.3%) with mean age 38.47 ± 10 years (range: 26–57 years) underwent surgery. At the end of follow-up period, a mean BMI reduction of 12.2 kg/m ^2^ kg/m ^2^ ( *p* < 0.001)] was observed. An excess weight loss (EWL) of 63% ( ± 34) was achieved at the end of the study. Additionally, total cholesterol and triglyceride levels decreased postoperative significantly. The main long-term complications were flatulence (60%), borborygmus (47.8%), mal odorous stool (30.4%), and diarrhea (21.7%). Revision rate was 4.34%. There were no cases with irreversible hepatic injury, deaths due to the surgery, or progressive renal failure.

**Conclusion** BIBP seems to be a safe, easily reversible, and one of valid therapeutic approaches in morbidly obese patients. BIBP has the potential to achieve durable weight loss and offers an improved quality of life.

Obesity is an increasing health conundrum and associated with diabetes mellitus, specifically type II, hypertension, coronary artery disease (CAD), cerebrovascular diseases, dyslipidemia, impaired quality of life, and premature mortality. In spite of rising attitude toward the problem, epidemiology of obesity, along with its related sequels continues to be at an alarming rate. 1 Several epidemiological studies have demonstrated that the prevalence of comorbidities decreases with weight reduction, so weight reduction maybe considered as an appropriate approach in treating comorbidities. 2 3

There are numerous methods in reducing weight but except for surgery, other methods are not completely successful. The long-term impacts of diet, exercise, and medical therapy are somewhat poor. 4 Many people selecting bariatric surgery have already applied numerous other weight loss methods, with little or no success. 5 The number of patients seeking bariatric surgery procedures are increasing and, to date, it is the only option resulting in substantial and durable weight loss. 4

Various surgical techniques have been developed to treat morbid obesity. Some of procedures have been evolved with time (e.g., jejunoileal bypass to biliointestinal bypass [BIBP]), whereas others have become obsolete. In general, the more complex the procedure, the better the results in terms of weight loss, but there are proofs that more complex procedures have higher morbidity and mortality rates. 6 Of all these techniques, biliaryintestinal bypass represents an appropriate model of massive and rapid weight reduction secondary to the induced malabsorptive state and, moreover, this technique is reversible. 7

The aim of this study was evaluating the long-term outcome, both advantages and complications of BIBP surgery in morbid obese patients.

## Materials and Methods

A prospective study was conducted in a private hospital from 2002 to 2016. Those patients not previously operated for morbid obesity were eligible for the study. Inclusion criteria were patients with BMI > 40 or patients with BMI 35 to 40 with at least one serious comorbid condition. Comorbid conditions were severe diabetes mellitus, hypertension, severe lipid disorders, severe sleep apnea, obesity-associated hypoventilation, and obesity-related cardiomyopathy. 8 Patients with the functional gall bladder who had not a biliary system surgery; who had refused to have a restrictive procedure with little success following medical treatment; and those with no drug, alcohol abuse, or psychopathology disorder, except for eating disorders were considered as eligible patients for this study. Patients who had severe altered self-body image, who were expected immediate weight reduction, and those unable to follow periodic medical controls were excluded from the study.

Anthropometric measurements, such as weight, height, and body mass index (BMI) defined as weight (kg)/height 2 (m ^2^ ), as well as past medical history, clinical presentation, preoperative workup were collected.

BMI, weight, concentration of blood, lipids and glucose, hepatic transaminases, postoperative changes in terms of cholelithiasis and urolithiasis, depression, anxiety, and body image dissatisfaction were gathered in follow-up visits. All the patients who had assigned consent form to undergo BIBP were evaluated by a multidisciplinary team (a surgeon, an intern, and a nurse). All the patients were operated through open technique.

After informing patients regarding BIBP surgery and related complications, a written informed consent was obtained from all patients.

### Surgical Technique

Section of the jejunum 30 cm from the Treitz ligament and of mesentery was made by linear stapler. The cholecysto–jejunal anastomosis was completed with hand sewn monofilament. A side-to-side anastomosis between the proximal jejunum and the last 50 cm of the ileum was created by firing a 60 mm linear stapler. On the excluded ileum an antireflux valve system was hand-sutured. 7 9 10

### Statistical Analysis

Statistical analysis was performed with SPSS (version. 16. for Windows). Results are expressed as mean ( ± SD) or number (%). Differences were considered statistically significant when *p* -value was less than 0.05.

## Results

**T** wenty-three patients, 7 (30.4%) males and 16 (69.5%) females, were underwent the BIBP surgery with the mean age of 38.47 ( ± 10) years. The mean follow-up time for all 23 patients was 89 ( ± 54) months (range: 73–108 months).

Mean body weight before the surgery was 125 ( ± 15) kg, while final weight was 86.2 ( ± 27) kg ( *p* < 0.001; Table 1 ). No significant differences were observed in weight reduction between female and male patients ( *p* < 0.397). Long-term mean weight loss was significant ( *p* < 0.001). Ninety-five percent of mean weight reductions was occurred during the first 2 years after surgery. Baseline BMI was reduced significantly from 44.9 ( ± 9.6) kg/m ^2^ at first to 39.2( ± 8) kg/m ^2^ at the end of follow-up period. Long-term changes of mean BMI was 12.2 kg/m ^2^ ( *p* < 0.001).

**Table 1:** Modified BMI, weight, FBS, AST, ALT, total Cholesterol, and Triglyceride

Parameters	Before BIBP mean ( ± SD)	After BIBP mean ( ± SD)	*p* -Value a
BMI (kg/m ^2^ ) male female	44.9 ( ± 9.6) 46.5 ( ± 14) 43.5 ( ± 5)	39.2 ( ± 8) 34.2 ( ± 11) 33.4 ( ± 4)	< 0.001
Weight (kg) male female	125 ( ± 15) 130 ( ± 24), 122 ( ± 12)	86 ( ± 27) 89.1( ± 10), 84.3( ± 32)	< 0.001
FBS (mg/dL)	95.2 ( ± 17.8) (range: 69–186)	89.6 ( ± 21.9) (range: 71–169)	0.125
AST (IU/L)	23.2 ( ± 17.2) (range: 11–74)	29.5 ( ± 13) (range: 9–41)	0.225
ALT (IU/L)	26.9 ( ± 11.9) (range: 14–69)	29.6 ( ± 17.2) (range: 9–50)	0.304
Total Cholesterol (mg/dL)	189.5 ( ± 53.9) (range: 97–368)	156 ( ± 31.9) (range: 93–203)	< 0.025
Triglyceride (mg/dL)	156.2 ( ± 39.7) (range: 88–421)	128 ( ± 17.2) (range: 80–234)	< 0.011

Abbreviations: ALT alanine aminotransferase; AST, aspartate aminotransferase; BIBP,; BMI, body mass index; FBS, fasting blood sugar. .

a *p* < 0.05 was statistically considered significant.

Prior to the surgery, mean cholesterol and triglyceride concentrations were 189.5 ( ± 53.9) mg/dL and 156.2 ( ± 39.7) mg/dL, respectively ( Table 1 ), and after the surgery, they were decreased to 156 ( ± 31.9; *p* < 0.025) and 128 ( ± 17.2; *p* < 0.011), respectively. Fasting blood sugar (FBS) level was reduced from 95.2 ( ± 17.8) mg/dL (range: 69–186 mg/dL) to 89.6 ( ± 21.9) mg/dL (71–169 mg/dL). At the end of follow-up period, two (66.6%) of three diabetic patients were still diabetic ( *p* < 0.99; Table 2 ). Liver function test (LFT) of all participants were transiently raised but the difference was not statistically significant ( Table 1 ). Before surgery, there were seven (30.4%) hypertensive patients, and at the end of study five (21.7%) subjects remained hypertensive ( *p* = 0.625).

**Table 2:** Remission of comorbidities following BIBP

Comorbidity	Before BIBP *n* (%)	After BIBP *n* (%)	*p* -Value a
Type 2 diabetes	3 (13.0)	2 (8.6)	0.99
Arthralgia biliointestinal bypass	13 (56.5)	7 (30.4)	0.625
Hypertension	7 (30.4)	5 (21.7)	0.625
Exertional dyspnea	16 (69.5)	2 (8.6)	0.001
low back pain	19 (82)	4 (17.3)	0.001
fatty liver disease	8 (34.7)	3 (13.0)	0.180
body image dissatisfaction	23 (100)	7 (30.4)	0.001

Abbreviation: BIBP, biliointestinal bypass.

a A *p* -value less than 0.05 was considered statistically significant.

The number of cases who suffered from chronic low back pain was reduced from 19 (82.6%) to 4 (17.4%; *p* < 0.001). Sixteen (69.5%) patients were affected by respiratory problems. At the end of follow-up period two (8.6%) of the patients remained symptomatic ( *p* < 0.001).

A total of 23 participants (100%) have reported symptoms of anxiety, depression, declined health-related quality of life, or body image dissatisfaction but at the end of follow-up period seven cases (30.4%) experienced such problems ( *p* < 0.001).

Of the 23 (100%) participants who underwent ultrasound studies, 2 (8.6%), 2 (8.6%), 5 (21.7%), 3 (13%) had developed renal stones, gall stones, first grade fatty liver, and second grade of fatty liver, respectively ( Table 2 ).

There was no mortality after this type of surgery. Postsurgery adverse outcomes ( Table 3 ) were as follows: flatulence (60%), borborygmus (47.8%), foul-smelling stool (30.4%), diarrhea (21.7%), fissure (17.4%), and hemorrhoids (13%).

**Table 3:** Complications following BIBP

Complication	*n* (%)
Flatulence	14 (60)
Borborygmus	11 (47.8)
Foul-smelling stool	7 (30.4)
Diarrhea	5 (21.7)
Fissure	4 (17.4)
Hemorrhoids	3 (13)

Abbreviation: BIBP, biliointestinal bypass.

None of patients developed any major complication but three (13%) of whom developed surgical site infection and two (8.6%) of whom developed atelectasis improved by conservative techniques.

None of patients (0.0%) developed liver or kidney failure. A reassessment rate of 4.34% was reported.

## Discussion

Biliointestinal bypass (BIBP) is a malabsorptive technique of surgery in which the small bowel is excluded from food transit. 7 The cholecystojejunal anastomosis facilitates bile transit into the loop and reduces bile absorption. 4 Overall, BIBP affects the enterohepatic axis function by diverting food away from the proximal gastrointestinal tract, delivering incompletely digested nutrients to the ileum. 4 5 7

In here, the long-term changes of mean weight and mean BMI were statistically significant ( *p* < 0.001). Moreover, a noticeable mean EWL of 63% (±34) was found, so 95% of mean weight reduction of patients occurred during the first 2 years following the surgery. Both cholesterol and triglyceride persistently reduced. Lubrano et al (2010) found that patients underwent BIBP demonstrated a significant reduction in total cholesterol (Tot-C), low-density lipoprotein cholesterol (LDL-C), and high-density lipoprotein cholesterol (HDL-C), as well as BMI, total fat mass (FM), triglycerides, BP, and inflammation markers. 4 Andersen et al (1988) have reported an impressive weight loss within the first 2 years after bypass. 11

Exciting outcomes of bariatric surgery are the reduced severity and, in some cases, remission of type II diabetes. Interestingly, many patients became euglycemic prior to weight loss. The procedure (BIBP) that expedite nutrient supply to the lower gastrointestinal tract appear to be especially promising in this respect. In our study, three patients (13%) had diabetes mellitus, 4 5 12 13 14 then it is important to be noted that blood sugar control was not significant after BIBP. Remission of type II diabetes was established in one (33.3%) of three (100%) patients who underwent BIBP ( *p* < 0.999) but long-term changes of mean FBS was not significant ( *p* = 0.125).

Several studies have demonstrated long-term improvements of hypertension following bariatric surgery. 4 7 Mason et al (2003) have demonstrated that in return for 10 kg weight loss both systolic and diastolic blood pressures will be decreased to 10 and 20 mm Hg, respectively. 15 Our study did not show such important changes, maybe due to inadequate population of hypertensive cases ( *p* = 0.625).

Other severe obesity-related comorbidities which are remission with weigh loss are obesity hypoventilation syndrome (OHS) and asthma. A great improvement in respiratory problems was reported among our participants ( *p* < 0.001; Table 2 ).

With respect to surgical procedures, most of the severe complications arise from bacterial overgrowth in the bypassed or blind segment of the small intestine. Some studies pointed out the absence of bacterial overgrowth and accumulation of toxic materials which decreases risk of liver fibrosis and cirrhosis in BIBP. In our study, no patients had liver or kidney failure. Lubrano et al reported that all BIBP patients had transient (3–6 months) hepatic alterations. As expected, all of our participants experienced transient elevation of hepatic transaminases but was not statistically significant 4 5 6 16 17 ( Table 1 ).

Development of gallstone is a complication related to postoperative weight loss. Yet, because cholelithiasis occurs in about one-third of patients who undergo bariatric surgery, it deserves attention. 4 5 The incidence of gallstones following bariatric surgery varies from 22 to 71%. 18 Gallstone and sludge formation has been reported in 8.7% of our participants. Only one patient had gallstone at the end of study ( *p* = 0.625).

Weight loss resulted from bariatric surgery has improved the severity of lower back pain and as well as overall functionality. 5 19 20 Our study showed a significant decrease in number of patients affected by chronic low back pain after surgery ( *p* < 0.001; Table 2 ). As expected, it appears that BMI and weight changes were remarkable predictors of pain abatement.

Arthralgia was improved in 7 of 13 patients who underwent bypass surgery but the difference was not statistically significant ( *p* < 0.625). Certainly owing to irreversible nature of osteoarthritic changes.

Numerous studies have described significant improvements in psychosocial functioning following bariatric surgery. 21 22 Our current investigation showed considerable decrease in psychic distress, anxiety, and body image dissatisfaction ( *p* < 0.001), as depicted in Table 2 . Finally, a reconsideration rate of 4.34% was reported.

Diarrhea is a drawback in all types of small intestinal bypass operations. Further review in literature has been suggested that the most common complications in short-term period are diarrhea (15%) and anal problems (62.5%), such as inflammation, hemorrhoids, and anal fissures. 4 6

Even though bariatric surgery is generally safe, there is always a little risk of death. Death occurs in approximately 0.15 to 0.64% of patients who have undergone bariatric surgery. 5 Mortality rate varies somewhat by surgery type.

In our study, there was no mortality report after BIBP surgery.

## Limitation

A major limitation for the current study is small sample size, so future studies are needed to evaluate more patients.

## Conclusion

BIBP seems to be a safe, easily reversible therapeutic approach in morbidly obese patients. It induces an effective and durable reduction of body weight and some obesity-related comorbidities and gives improvement in quality of life. Most BIBP adverse events do not require hospitalization.
